# Eating Difficulties among Older Adults with Dementia in Long-Term Care Facilities: A Scoping Review

**DOI:** 10.3390/ijerph181910109

**Published:** 2021-09-26

**Authors:** Dukyoo Jung, Kyuri Lee, Jennie C. De Gagne, Minkyung Lee, Hyesoon Lee, Leeho Yoo, Sarah Won, Eunju Choi

**Affiliations:** 1 College of Nursing, Ewha Womans University, Seoul 03760, Korea; dyjung@ewha.ac.kr (D.J.); kyurilee2501@gmail.com (K.L.); sunnysoonlee@nate.com (H.L.); haha_riho@naver.com (L.Y.); van927@naver.com (S.W.); 2 School of Nursing, Duke University, Durham, NC 27710, USA; jennie.degagne@duke.edu; 3 Department of Thoracic Surgery, Mount Sinai Hospital, New York, NY 10029, USA; mkmk8888@naver.com

**Keywords:** aged, dementia, eating behavior, nursing homes, review

## Abstract

This paper reports a scoping review of the literature on eating difficulties among older adults with dementia in long-term care facilities to identify key concepts, methods of measuring outcomes, interventions, and related factors. A scoping review was performed using the bibliographic databases PubMed, CINAHL, PsycINFO, and Cochrane Library. A combination of keywords and subject headings related to eating or feeding difficulties was used. Inclusion criteria were limited to materials published in English. A total of 1070 references were retrieved, of which 39 articles were selected after applying the inclusion and exclusion criteria. Articles that met the criteria were published between 1987 and 2020. “Eating disabilities” have been defined as problems related to choosing food and/or the ability to get food to one’s mouth, chew, and swallow. Interventions for eating difficulties described in the literature include spaced retrieval training, Montessori training, and feeding skill training. Intrapersonal, interpersonal, and environmental factors related to eating difficulties were identified. This scoping review will provide direct care workers, nursing educators, and administrators with an overview of eating performance and a broad understanding of eating difficulties for older adults with dementia in long-term care facilities.

## 1. Introduction

Worldwide, around 50 million people have dementia, a major cause of disability and dependency among older people [1]. The total number of people with dementia is projected to reach 82 million in 2030 and 152 million in 2050 [1]. Dementia is a disease that causes progressive deterioration affecting cognitive function, physical function, language, and memory [2]. In particular, the physical function of older adults with dementia gradually decreases until they need to enter long-term care facilities [3]. According to the 2018 Health Insurance Review & Assessment, over 15% of older adults with dementia have been institutionalized in Korea [4]. In a previous study, the majority of older adults with dementia were found to die in long-term care facilities [5]. The proportion of medical expenses for dementia is significant and has become a social and economic burden: in 2015, the total global societal cost of dementia was estimated to be US$818 billion, equivalent to 1.1% of the global GDP [1].

The majority of older adults with dementia have been found to have nutritional imbalances [6], and nutritional deprivation is commonly reported in individuals institutionalized for 8 or more years in long-term care facilities (LTC) [7]. Additionally, a study found that 62.8% of older adults in LTC facilities have severe cognition impairment, with 32.2% reliant on dependent eating [8]. As dementia progresses, the ability to swallow [9], remember to eat, recognize food, and eat independently becomes impaired, and the need for constant intervention and support during meals increases [10]. Changes in movement (ability “to eat without dropping food”), concentration (ability “to maintain attention on the meal”), and safety (ability “to swallow without choking, with no change in vocal quality after eating”) have been significantly associated with mortality risk ([11] p. 162), impaired functionality, malnutrition, and respiratory infections [9,10]. Residents’ ability to eat independently is lost, increasing their dependency on others to provide feeding assistance to meet their nutritional needs [12,13]. Abnormal eating behaviors, changes in eating and dietary habits, and serious feeding difficulties are present in most people with dementia [13]. Eating difficulty is a combination of behavioral and psychological symptoms of dementia (BPSD), evidenced by vital signs such as the inability to use eating utensils properly, leaving meals unfinished, eating non-food materials, and hunger. Eating difficulties are manifested in various ways (e.g., swallowing a too-large lump of food, inability to begin eating) depending on which function (e.g., memory, attention, judgment) is declining [14]. Residents with dementia in long-term care facilities are at greater risk of low food intake compared to residents who can engage in independent feeding [10]. It is important to note that eating difficulty may not only be a consequence of progressive metabolic or neurochemical abnormalities but also the result of insufficient caregiving [13]. The reality is that dietary assistance is largely based on the habits and experiences of dietary assistants [15,16].The purpose of this review was to (1) identify key concepts of “eating difficulties,” (2) investigate the types of research that have been conducted on eating difficulties, and (3) examine the method of measuring outcomes of eating difficulties for older adults with dementia in long-term care facilities (LTC) and nursing-homes (NH).

## 2. Materials and Methods

### 2.1. Study Design and Methodology

To examine the key concepts of eating difficulties among older adults with dementia residing in long-term care facilities, a scoping review was performed following the framework developed by Arksey and O’Malley [17] and recently revised by Levac et al. [18]. The scoping review method is utilized to determine the extent and nature of the evidence available on a topic [19]. Contrary to systematic reviews, which strive to answer a precise question, the scoping review methodology has the capacity to support a knowledge synthesis that addresses an exploratory research question, allowing researchers to map a conceptual framework, different types of evidence, and gaps in the research field [20]. To facilitate this research scope and its objectives, the current review utilized a Population–Concept–Context model, wherein the population was older adults with dementia and their direct care workers, the concept was feeding or eating difficulties, and the context was long-term care facilities where older adults with dementia reside. For the present scoping review, the following steps were performed: (1) research question formulation; (2) identification of relevant studies; (3) selection of relevant studies; (4) data charting; (5) collection, summary, and reporting of findings [20,21]. 

### 2.2. Research Question

The following research question was formulated: What studies have been conducted on “eating/feeding difficulty” among older adults with dementia living in long-term care facilities?

### 2.3. Search Strategy

The search strategy was adopted to identify the published and unpublished literature following the three-step approach developed by The Joanna Briggs Institute (JBI) [22]. First, preliminary computerized searches of two electronic databases in the Cumulative Index to Nursing and Allied Health Literature (CINAHL) and PubMed were performed to select accurate search terms. The authors initially reviewed the titles, abstracts, and index terms used to describe the captured articles. Through the process of checking and adding various search terms with similar meanings, the accuracy of search term selection was improved. Based on the preliminary search, the literature search was conducted in PubMed, CINAHL, PsycINFO, and Cochran library. No date limits were placed on the search, but the results were limited to English-language materials. For a broad search, “eating” and “feeding” were used as key words, and “feedings/feeds” were also included. The search terms referring to older adults with dementia combined "geriatric/aged/older/elderly/senior” and “dementia/s/” with the (and) operator. Facilities were searched using “facility*/long term/institutional/nursing home*”. The following specific search terms were used in the searched literature: Eating disabilities, Food intake difficulties, Eating behavior difficulties, Eating abnormality, Eating disorder, Eating difficulties, Eating disturbance, Mealtime behavioral challenges, Poor food in-take, and Problem feeding behavior. A full search strategy for each database is provided in Table S2. Studies were screened using a four-stage process to determine inclusion or exclusion for review: (1) title scanned, (2) abstract viewed, (3) full text viewed, and (4) reference lists of the article selected in the second phase checked. Full-text studies that did not meet the inclusion criteria were excluded, and the reasons for exclusion were noted through discussions between the reviewers. Two researchers independently reviewed the selected abstracts, and only relevant studies were chosen by reviewing the full text. If there was no consensus of opinion, an attempt was made to ask a third reviewer for the final selection, but there was no third-party review because the literature selection was consistent. This search was conducted from October to December 2020. After completion of the second review and a full-text screening of search results, a third-phase review of reference lists was conducted.

### 2.4. Study Selection

The search results were exported to a bibliography software (EndNote) and systematically stored in groups linked to the database of origin. Duplicates were removed and the remaining studies were then exported to an Excel file containing the extraction fields. Studies were assessed against the inclusion/exclusion criteria listed below. 

Inclusion criteria:

1. Incorporated contents were those related to the current research topic, including “eating disabilities”, “food intake difficulties”, “eating behavior difficulties”, “eating abnormality”, “eating disorder”, “eating difficulties”, “eating disturbance”, “mealtime behavioral challenges”, “poor food intake”, and “problem feeding behavior”;
2. Participants were older adults (65 years or older) with dementia;
3. Participants resided in long-term care facilities or nursing homes;
4. Articles written in English.

### 2.5. Data Extraction

**Figure 1:**
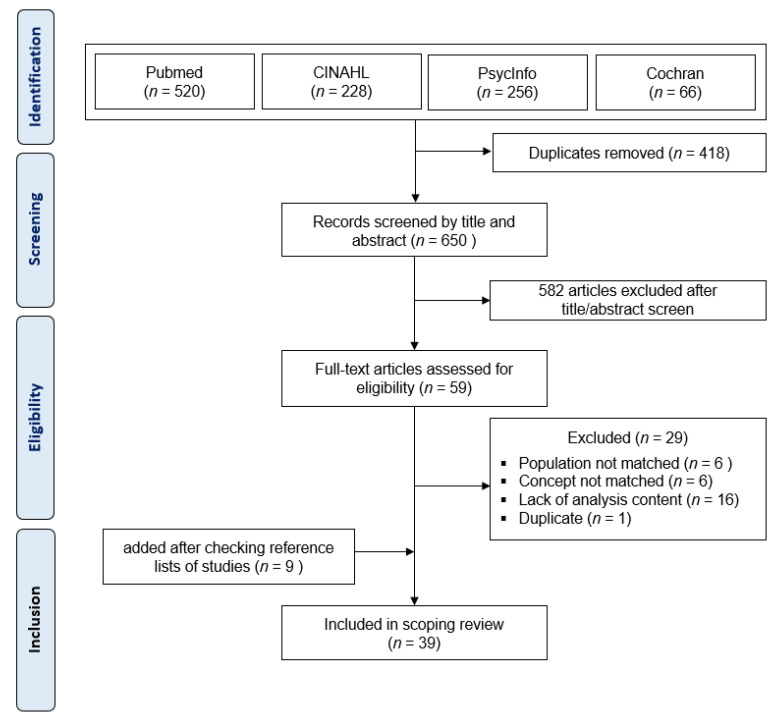
Flowchart of the selection process.

A total of 59 retrieved articles had their titles and abstracts reviewed using the study eligibility derived from the inclusion criteria. Nine studies were added from the references of the adopted articles, resulting in full copies of 39 studies for appraisal and final review (Figure 1). 

To further standardize our data extraction and charting process, we used an ad hoc grid in Microsoft Excel to collate the details from each paper that are relevant to our review aims: (a) citation details, (b) year, (c) country, (d) aims, (e) design, (e) concept, (f) measurements, (g) interventions, and (h) associated factors.

## 3. Results

### 3.1. Overview

As shown in Figure 1, initially, there were 650 potentially relevant studies. Of the 59 articles that were fully reviewed by the authors, 29 (49.1%) were excluded for the following reasons: (a) they did not match the population of older adults with dementia (*n* = 6), (b) the concept of feeding and eating was absent (*n* = 6), (c) analysis content was lacking (e.g., “Eating difficulties are not the main content”) (*n* = 16), and (d) content that could not be found in an academic journal was checked in full text and removed as a duplicate (*n* = 1). A total of 39 studies published between 1989 and 2020 met the inclusion criteria (Table 1 and Table S1).

### 3.2. Study Characteristics

The 39 reviewed articles had a total of 7043 study participants. The included studies comprised intervention studies (*n* = 8), observational studies (*n* = 17), a qualitative study (*n* = 1), mixed method studies (*n* = 3), literature reviews (*n* = 5), systematic reviews (*n* = 4), and a scoping review (*n* = 1). Research has been conducted in various countries such as Taiwan (*n* = 11), the Unities States of America (*n* = 7), Italy (*n* = 2), South Korea (*n* = 3), the Netherlands (*n* = 1), Canada (*n* = 1), Japan (*n* = 1), Spain (*n* = 1), and Sweden (*n* = 1). The studies were conducted between 1989 and 2020. Regarding the explanation of each article, the author, publication year, study aim, study participants, measurements, and outcomes were reviewed (Table 1). The eating disabilities of older adults with dementia were described as eating ability, feeding difficulty, eating difficulty, and mealtime feeding behavior.

### 3.3. The Concept of Feeding Difficulties

In the articles, “eating difficulties” and “feeding difficulties” are used interchangeably. Although “eating” and “feeding” have different meanings, in previous studies, the distinction between “eating “and “feeding” in the elderly with dementia is ambiguous. This is because “feeding” of the elderly with dementia could also be considered “eating.” Therefore, in this study, “feeding” and “eating” among the elderly with dementia was included.

In an article by Chang and Roberts [54] analyzing the concept of “feeding difficulties,” they acknowledged that eating and feeding are somewhat similar, but differ in terms of the extent to which difficulties affected caregivers’ abilities to feed older adults. Thus, the attribute of “feeding difficulties” is defined as specific problems experienced by caregivers while feeding patients with dementia. Feeding difficulties include initiating feeding tasks, maintaining attention on the task of feeding, difficulty getting food into the mouth, difficulty chewing food, and difficulty swallowing food. 

### 3.4. The Outcome Measurements

Feeding difficulties were measured using standardized instruments applied to older adults with dementia or their caregivers. For older adults with dementia, eating difficulties were mainly measured using the Edinburgh Feeding Evaluation in Dementia (EdFED) [59,60,61,62,63,64,65], the EdFED has been translated into Chinese [66] and Italian [67]. Additionally, the Eating Behavior Scale (EBS) [68] was used to measure the eating behaviors of older adults with dementia. These results are due to EdFED’s confidence in and validation of the elderly in long-term care hospital facilities [62,63]. Further, EBS is an observation-based, easily applicable tool that does not impose an additional burden on patients and can be easily used by anyone [68]. For caregivers, questionnaires concerning knowledge/behavior/attitudes [23,24] related to feeding patients with dementia were mostly applied (Table 1).

### 3.5. Interventions Related to Eating Behaviors in the Studies

We reviewed 10 studies (8 intervention and 2 mixed-methods studies) on eating behaviors among older adults with dementia that included caregivers as well as older adults with dementia as the study participants. The intervention types consisted of spaced retrieval training, Montessori, combined SR and Montessori, feeding skills training, and web-based dementia feeding skills training (Table 2). The majority of the sample sizes were 51–100. The intervention length per session was under 60 min, and the intervention duration was more than 8 weeks. The intervention outcome measures for older adults with dementia were comprised of the feeding difficulty index, feeding behavior, total eating time, food intake/eating amount, nutritional status, self-feeding frequency, and body weight. Additionally, the intervention outcome measures for caregivers consisted of knowledge, attitudes, behavior, intention, physical assistance, verbal assistance, the number of residents fed by caregivers, and time spent providing meal assistance (Table 2).

### 3.6. Associated Factors

The analysis of related factors of eating difficulties among older adults with dementia residing in long-term care facilities is shown in Table 3; this table classifies the variables related to eating difficulties identified in the 13 observational studies included in this review into intrapersonal, interpersonal, and environmental factors. A total of 17 variables were identified: 9 intrapersonal factors, 2 interpersonal factors, and 6 environmental factors. Regarding frequency of mentioned items, the intrapersonal factor “cognitive function” was the most common; for environmental factors, “physical environment” was the most common; for interpersonal factors, “close relationship with family” was the most common. These were, therefore, identified as factors that can significantly affect eating behavior.

## 4. Discussion

This scoping review examined the literature on eating difficulties and feeding difficulties of older adults with dementia residing in long-term care facilities, shedding light on the eating difficulties within this population. Existing studies have identified the concept of eating difficulties, outcome measures, intervention studies, and related factors. 

We found that previous studies have used the terms “feeding difficulties” and “eating difficulties” interchangeably. For instance, according to Melloni’s Illustrated Medical Dictionary, “feeding” and “eating” are defined differently: “feeding” means giving food or administering nourishment [69], whereas “eating” refers to taking, chewing, and swallowing food. Further, “eating difficulties” has been used to describe the quality and quantity of food a patient chooses to eat, reflecting only the food consumed, and not that which has been fed or not eaten [70]. In research in the nursing field, the terms “eating” and “feeding” have been used synonymously, making it difficult to ascertain whether the older adults with dementia themselves or their caregivers have assumed the responsibility of feeding. In the previous literature, the terms “eating difficulties” and “feeding difficulties” have been used without clear distinctions [54].

Feeding difficulties are defined as specific behaviors produced while the caregiver is feeding an individual. In their study [54], Chang and Roberts defined feeding and eating characteristics, acknowledging that eating and feeding are somewhat similar, but that feeding difficulties refer to the problems experienced by caregivers while feeding older adults with dementia, rather than the problems experienced by the older adults who are being fed. Feeding difficulties include caregivers’ efforts to (a) get food into the individual’s mouth and (b) help the individual overcome or compensate for problems related to chewing and swallowing. Additionally, feeding difficulties may involve needing to help the individual initiate and maintain attention to the feeding task. Physical (i.e., poor motor control, cognitive impairment, perceptual deficit), psychological (depression, anxiety), and social factors (social interaction) were defined as contributors to feeding difficulties. Furthermore, weight loss, malnutrition, and aspiration were mentioned as consequences of feeding difficulties [54]. The EdFED and EBS are the main standardized measurements for analyzing eating difficulties in older adults with dementia (Table 1); however, the EdFED is limited because it covers only some behaviors of older adults with dementia [59,60,61,62,63,64,65]. The EBS was developed to measure functional ability during meals and includes meal and eating behaviors [68]; thus, EdFED and EBS are limited in their ability to explain dementia-related behaviors during mealtimes. It is suggested that the antecedent factors, environmental factors, and outcomes should be included in measurements because the eating behaviors of older adults with dementia are likely to be influenced by these factors [54]. Moreover, risk factors of eating difficulties in older adults with dementia, such as dysphagia, difficulty using utensils, positioning for meals, level of feeding assistance needed, and agitation, should be included in the measurements [49]. It is highly recommended that, in future research, comprehensive and appropriate measurements should be developed for the detailed measurement of eating difficulties in older adults with dementia.

Interventions for eating difficulties in older adults with dementia were classified into studies targeting older adults with dementia and their caregivers. Spaced retrieval (SR) training, Montessori, and Montessori combined with SR interventions were conducted on older adults with dementia. SR is a step-by-step training method to improve eating memory (and, thus, the eating process and eating behavior) through interval training [25,29]. Montessori education is a method for treating residents with dementia in clinical practice. The Montessori education program developed by Camp [71] has been used [25,26] for education and focuses on hand–eye coordination, scooping, pouring, and squeezing related to eating difficulties. This type of intervention program focuses on cognitive ability because cognitive impairment in older adults with dementia can lead to difficulties with independent eating, resulting from loss of perception of the need to eat. This method is also employed when residents have difficulty using eating utensils and performing movements due to apraxia [10]. 

As the research on eating difficulties in older adults with dementia progresses, an educational method that combines SR training with Montessori training or Errorless learning is being implemented. As errorless learning increases memory efficiency by removing errors that may occur in the first learning stage of information [72], it is frequently applied and used together with SR [73]. As such, training methods are changing because their effects can be improved when the two methods are combined and complement each other [28,73]. All interventions for older adults with dementia included in this study focused on cognitive intervention. However, environmental factors also influence the eating difficulties experienced by older adults with dementia, so more studies on environmental change interventions are needed.

This review also examined studies focusing on caregiver interventions for eating difficulties in older adults with dementia; these were mainly studies based on behavior modification for skill training for caregivers assisting older adults with dementia during mealtime. Previous studies were conducted as a direct education program for caregivers, but the recent development of new mobile media, Wireless Fidelity (WIFI), and Artificial Intelligence (AI) technologies, through smartphones and tablets [74] has allowed for Web- or mobile-based programs using these technologies. In the studies of caregivers included in this review, regarding eating difficulties [15,23,24], it was found that the length of mealtime [23,24] and ingested meal amount [15] increased in older adults with dementia. This result may be due to the fact that improved caregiver knowledge increased their awareness of eating disorders in older adults with dementia [23,24]. “Increase in mealtime” means that caregivers provided longer mealtimes to older adults with dementia, giving them longer periods to eat [23]. Furthermore, improvements in knowledge, behaviors, and attitudes in caregivers through training were inconsistent across studies. In some cases, knowledge, behavior, and attitude all improved [23], but in others, caregiver knowledge and behavior improved while attitude did not [24]. This inconsistency may have been due to the differences in the intervention periods, as sufficient time is needed for knowledge acquisition to be reflected by changes in attitudes and behaviors [48,75]. In this regard, the use of web or mobile education to teach at an individual’s speed during their free time might be useful for encouraging self-efficacy [15,76] among caregivers. 

This review found that most of the interventions for care providers were conducted for less than 8 weeks, with sessions lasting more than 60 minutes. For older people with dementia, more than 8 weeks of education were generally conducted, with sessions lasting less than 60 minutes. It is necessary to consider a sufficient intervention period when designing an intervention. Based on previous studies, it takes at least 6 to 12 weeks to confirm an intervention’s effect on eating difficulties [15,77].

Factors associated with eating difficulties were classified into three-level domains based on the Social Ecological Model [8,78]: intrapersonal, interpersonal, and environmental. The factors observed in this review were primarily categorized at the intrapersonal and environmental level. Intrapersonal factors associated with reduced eating performance of residents included declined cognitive [7,8,36,37,38,39,42] and physical functions [35,37,40], long periods of residence in LTC [7,40], female sex [35,44], old age [35], presence of comorbidities [36], longer illness duration [59], increased usage of medications [19], and presence of dysphasia [38]. It remains unclear whether age and sex significantly affect eating ability, as they were not identified as significant factors in some studies. Among the multiple individual factors identified, cognitive and physical function were most frequently shown to be strong contributing factors, supporting the need for strategies that prevent functional decline. 

Environmental factors significantly associated with the eating difficulties of residents with dementia include the absence of supervisory staff [7,35], non-public eating places [7,34], physical settings (louder sounds and low light level) [41], diet type (soft and liquid food) [7], longer mealtimes [41], and eating without other residents [44]. A dining area is a place for social interaction, and physical environmental factors are important for residents with dementia, who are often easily distracted by perceptual surroundings [79]. As unmeasured aspects are certainly present in these environmental factors, more studies stratifying individual variables, including cognitive function, severity of functional impairment, and other intrapersonal or interpersonal factors, are crucial for producing more robust and reliable findings. Residents eating in their room instead of a public area, for example, may have severe cognitive changes or limited mobility, as the authors have concluded [7]. 

Significant interpersonal factors, on the other hand, include close relationships with family [35,44] as well as caregivers’ visual and physical assistance during mealtime [63]. While direct caregivers are in a strategically essential position to assist institutionalized older adults with dementia and high dependence to carry out activities of daily living, and interpersonal factors are relatively modifiable compared to the individual characteristics [41,80], there is a paucity of studies reporting the influences of a caregiver’s characteristics. Further studies are, therefore, needed to explore the relationships between eating performance and various caregiver variables such as professional knowledge or competence, skills, and feeding assistance time. Additionally, a research gap remains regarding the specific policy factors that influence eating difficulties. It is recognized that laws and institutional policies influence the health of older adults with dementia at both the population and individual levels. In particular, legislation on minimum staffing ratios and the proportion of nurses at LTC facilities may be associated with the quality of care and resident health outcomes [81]. Future research should aim to identify and analyze various existing policies among different countries and institutions, as well as their effects on residents’ eating difficulties. Moreover, further longitudinal studies are needed to better understand changes in eating difficulties throughout the course of dementia progression.

This review comprehensively analyzed the trends and status of eating difficulties for older adults with dementia. Concepts of eating difficulties, interventions related to eating behaviors, and feeding factors affecting eating disorders were considered from various angles. Accordingly, the results of this study may influence the work of health care workers, policy makers, and researchers studying eating difficulties in long-term care facilities.

### Limitations

This review has limitations. The definition of eating difficulties is not combined with feeding difficulties, which limits how we can generalize the meaning of eating difficulties for older adults with dementia. Additionally, this research is limited to English-language publications. The EdFED version has been translated into Chinese, Italian, and Korean; thus, we expect that there may be other relevant eating difficulties studies written in other languages. For future studies, eating difficulties should be conceptualized and differentiated from feeding difficulties. Additionally, comprehensive measurements for eating difficulties among older adults with dementia should be developed.

## 5. Conclusions

The purpose of this study was to synthesize previous studies on eating difficulties of older adults with dementia in long-term care facilities. A total of 39 studies were selected, and it was found that about 40% of the literature consisted of observational studies. This review identified the concepts of eating and feeding difficulties and reviewed the associated factors as well as related measures and interventions. EdFED and EBS were mainly used as eating behavior evaluation tools; intervention methods tend towards a form of intervention that combines the two methods. In addition, individual factors, interpersonal factors, and environmental factors contributing to the eating difficulties of the elderly with dementia were identified. The factors identified as having highest frequency related to eating difficulties were cognitive function and physical function under intrapersonal factors; close relationships with family or caregivers under interpersonal factors; and elements of the physical environment such as a tablecloth, illuminance level, and volume level under environmental factors. The findings of this scoping review will directly inform care workers and administrators in long-term care facilities, researchers, and educators.

## Figures and Tables

**Table 1:** Summary of included studies (*n* = 39).

Design	Author(s) (Reference Number)	Country	Aims	Population	Outcome Measurements
	Batchelor-Murphy et al. [15]	USA	To evaluate the feasibility of a web-based dementia feeding skills training program for nursing home staff	▪ 7 nursing assistant–dementia patient dyads -exp. (*n* = 4) -cont. (*n* = 3)	▪ EdFED ▪ Time spent providing meal assistance ▪ Meal intake
Intervention study	Chang et al. [23]	Taiwan	To evaluate the effectiveness of a feeding skills training program for nursing assistants	▪ 67 nursing assistants -exp. (*n* = 31) -cont. (*n* = 36) ▪ 36 nursing assistant–dementia patient dyads -exp. (*n* = 20) -cont. (*n* = 16)	▪ The Formal Caregivers’ Knowledge of Feeding Dementia Patient Questionnaire ▪ The Formal Caregivers’ Attitude toward Feeding Dementia Patient Questionnaire ▪ The Formal Caregivers’ Behaviors toward Feeding ▪ EdFED ▪ Total eating time ▪ Food intake
	Chang et al. [24]	Taiwan	To evaluate the effectiveness of a feeding skills training program for nursing assistants	▪ 67 nursing assistants -exp. (*n* = 31) -cont. (*n* = 36) ▪ 36 nursing assistant–dementia patient dyads -exp. (*n* = 20) -cont. (*n* = 16)	▪ The Formal Caregivers’ Knowledge of Feeding Dementia Patients Questionnaire ▪ The Formal Caregivers’ Attitude toward Feeding Dementia Patients Questionnaire ▪ The Perceived Behavior Control Scale ▪ The Intention Scale ▪ The Formal Caregivers’ Behaviors in Feeding Dementia Patients Observation Checklist ▪ Feeding during mealtime was observed
	Lin et al. [25]	Taiwan	To evaluate the effectiveness of a training protocol (Spaced Retrieval and Montessori-based activities) in decreasing eating difficulty	▪ 85 residents with dementia -exp. (SR) (*n* = 32) -exp. (Montessori) (*n* = 29) -cont. (*n* = 24)	▪ Chinese version of EdFED ▪ MNA (Mini-nutritional assessment) ▪ Observation (Eating time, Eating amount, Residents fed by caregivers, Physical assistance, Verbal assistance)
	Lin et al. [26]	Taiwan	To evaluate the effectiveness of a Montessori intervention for improving eating ability and nutritional status	▪ 29 residents with dementia -Montessori intervention sequence I (*n* = 15) -Montessori intervention sequence II (*n* = 14)	▪ Chinese version of EdFED ▪ EBS ▪ MNA ▪ Observation (Self-feeding frequency, Self-feeding time, Verbal assistance, Physical assistance, Residents fed by caregivers)
	Wu et al. [27]	Taiwan	To evaluate the long-term effects of the standardized and individualized spaced retrieval combined with Montessori-based activities on eating ability	▪ 61 residents with dementia -exp. (SR/EL group) (*n* = 32) -cont. (SR-only group) (*n* = 29)	▪ The proportion of each meal consumed
	Wu et al. [28]	Taiwan	To evaluate the effects of using accumulating cues in a spaced retrieval paradigm on recall performance, cognitive status, and food intake	▪ 90 residents with dementia -exp. (Montessori-based group) (*n* = 25) -exp. (Individualized group) (*n* = 38) -cont. (*n* = 27)	▪ Chinese version of EdFED ▪ Eating amount ▪ Body weight
	Hsu et al. [29]	Taiwan	To evaluate the effectiveness of spaced retrieval for improving hyperphagia	▪ 97 residents with dementia -exp. (*n* = 50) -cont. (*n* = 47)	▪ Dementia Hyperphagic Behavior Scale ▪ Food intake ▪ BMI
Observational study	Lee et al. [7]	Korea	To investigate factors associated with eating ability	▪ 149 residents with dementia	▪ MMSE-K ▪ Korean activities of daily living scale ▪ Eating Behavior Scale
	Liu et al. [8]	USA	To investigate the association between specific personal and environmental factors and eating performance	▪ 199 residents with dementia	▪ Using the single self-care ‘feeding’ item in the Barthel Index ▪ MMSE ▪ Using the single ‘chair sit-sitting balance’ item in the Tinetti Gait and Balance scale ▪ Physical Capability Scale (PCS) ▪ Cornell Scale for Depression in Dementia (CSDD) ▪ Cohen-Mansfield Agitation Inventory-short form (CMAI)
	Durnbaugh et al. [30]	USA	To present the Feeding Behaviors Inventory, an instrument designed to identify common mealtime feeding behaviors	▪ 20 residents with dementia	▪ Feeding behaviors inventory
	Berkhout et al. [31]	Netherlands	To investigate the cause of weight loss in nursing-home patients with dementia	▪ 514 residents above 65 years in nursing home -existing residents: (*n* = 250) -newly admitted: (*n* = 264)	▪ Nurses recorded the most important difficulties in self-feeding (choosing food, bringing food to the mouth, chewing, and swallowing)
	Amella [32]	USA	To predict how the quality of the interaction between care giver and care receiver influenced the proportion of food consumed	▪ 53 residents with late-stage dementia.	▪ Proportion of food consumed (weighing)
	Amella [33]	USA	To investigate factors regarding resistance behavior at meals	▪ 53 residents with dementia -resistors (*n* = 23) -acceptors (*n* = 30)	▪ EdFED-Q ▪ BMI ▪ Proportion of food consumed (weighing) ▪ Time taken to assist with meals
	Reed et al. [34]	USA	To investigate factors associated with low food and fluid intake	▪ 421 residents with dementia	▪ The Structured Meal Observations (SMO)
	Lin et al. [35]	Taiwan	To investigate the risk factors of low food intake	▪ 177 residents with dementia in LTC	▪ Chinese version of EdFED ▪ BMI
	Slaughter et al. [36]	Canada	To estimate the incidence and identify the predictors of eating disability due to dementia	▪ 120 nursing home residents	▪ Researchers observed residents’ loss of eating ability during meals; eating disability was defined as receiving physical assistance to put food into the mouth or not eating at all
	Chang et al. [37]	Taiwan	To investigate factors associated with feeding difficulty of individuals with dementia	▪ 93 residents with dementia	▪ Chinese version of EdFED
	Edahiro et al. [38]	Japan	To investigate factors affecting self-feeding	▪ 150 Alzheimer’s disease patients who were hospitalized in dementia ward	▪ Feeding Cycle Recording ▪ Eating-related BPSD item
	Hanson et al. [39]	USA	To describe quality of care for feeding problems in residents with advanced dementia, and probability and predictors of weight loss and mortality.	▪ 256 residents with dementia	▪ Quality of feeding assistance provided by staff ▪ Body weight loss ▪ Mortality
	Wu [40]	Taiwan	To explore the prevalence and predictors of hyperphagic behaviors	▪ 179 residents with dementia	▪ Hyperphagia questionnaire ▪ Cognitive abilities screening instrument ▪ Cohen-Mansfield agitation inventory ▪ Demographic characteristics
	Chang et al. [41]	Taiwan	To identify the best cutoff point for the Chinese Feeding Difficulty Index (Ch-FDI) and factors associated with eating behaviors	▪ 213 residents with dementia	▪ Chinese Feeding Difficulty Index ▪ EdFED
	Maria Perez-Sanchez et al. [42]	Spain	To evaluate the relation between altered eating behaviors/attitudes and nutritional status	▪ 139 residents with severe cognitive impairment	▪ The Blanford’s Aversive Feeding Behaviors Inventory ▪ The 26-item Eating Attitudes Test ▪ Dietary intake ▪ MNA
	Liu et al. [43]	USA	To examine the association of resident characteristics, staff mealtime assistance, and environmental stimulation with the pace of food intake	▪ 19 NA and 15 residents with dementia	▪ The pace of food intake recorded on video ▪ Level of Eating Independence (LEI) scale ▪ Staff mealtime assistance recorded on video
	Palese et al. [44]	Italy	To explore the influence of nursing home environment on eating independence	▪ 1027 residents with dementia	▪ Italian-validated version of EdFED
Qualitative study	Athlin et al. [45]	Sweden	To understand feeding problems in patients with severe dementia cared for in a task assignment system	▪ 15 patients with severe dementia and 45 caregivers who fed the 15 patients during the study period	▪ Researchers analyzed video-recorded meals for patients with severe dementia and interviews with caregivers who assist them.
Mixed methods	Chang et al. [46]	Taiwan	To investigate factors related to feeding difficulty that are shown in the interaction between nursing assistants and residents	▪ 48 residents with dementia and 31 nursing assistants	▪ Chinese version of EdFED ▪ Nursing assistant interview about feeding dementia residents
	Shinagawa et al. [47]	Japan	To develop a possible classification of eating-related problems	▪ 208 residents and patients	▪ Semi-structured systematic interviews with nurses stationed on Eating and Swallowing ▪ Mini-Mental State Examination (MMSE) ▪ Clinical Dementia Rating (CDR) ▪ Neuropsychiatric Inventory (NPI)
	Jung et al. [48]	Korea	To assess the feasibility and examine the preliminary effectiveness of a mobile application-based meal assistant training program for use by direct care workers	▪ 23 older adults with dementia-caregiver dyads	▪ Eating behavior scale (EBS)
Literature review	Keller et al. [49]		To present meal rounds as a potential intervention for identifying nutrition problems and specifically feeding, food texture, and mealtime behaviors.	▪ A previously published study on 37 residents	N/A
	Cleary [50]	Canada	To review the current approaches to manage feeding and swallowing disorders	N/A	N/A
	Aselage et al. [51]	USA, UK, Australia	To explore the state of mealtime difficulties; characteristics, measurements, related factors, and interventions for alleviating mealtime difficulties	N/A	N/A
	Chang et al. [52]	Taiwan, USA	To propose strategies for feeding patients that caregivers can use	N/A	N/A
	Cole [53]	USA	To explore interventions that can be undertaken to establish and maintain adequate nutritional intake	▪ 12 articles	N/A
Systematic review	Chang et al. [54]	Taiwan	To use concept analysis to identify characteristics of feeding difficulty and its antecedents and consequences that provide direction for assessment and management	▪ 71 articles	N/A
	Liu et al. [55]	USA	To evaluate the effectiveness of interventions on mealtime difficulties	▪ 22 intervention studies -2082 residents with dementia -95 professionals -85 long-term care facilities	N/A
	Liu et al. [56]	USA	To evaluate the effectiveness of interventions on eating performance	▪ 11 articles	N/A
	Fetherstonhaugh et al. [57]	Australia	To review the literature on strategies for promoting mealtime function in people with dementia living in residential aged care facilities and assess their effectiveness	▪ 20 articles	N/A
Scoping review	Palese et al. [58]	Italy, UK	To map the state of the research designed to maintain and/or promote independent eating	▪ 17 articles	N/A

exp. = experiment; cont. = control; NA = Nurse Assistant EdFED = Edinburgh Feeding Evaluation in Dementia; BMI = Body Mass Index; EBS = Eating Behavior Scale; MNA = Mini-Nutritional Assessment; MMSE = Mini-Mental State Exams; ADOD = Alzheimer’s Disease and Other types of Dementia; NPI = Neuropsychiatric Inventory; SMO = the Structured Meal Observation tool; CDR = Clinical Dementia Rating.

**Table 2:** Interventions related to eating behaviors in the intervention studies (*n* = 10).

Variables	Categories	*n*	Reference No.
Participants	Caregiver	5	[15,23,24,46,48]
	Older adults with dementia	5	[15,25,26,27,28]
Intervention type	Spaced retrieval (SR) training	2	[25] ^1^, [29]
	Montessori	2	[25] ^1^, [26]
	Combined with SR	2	[27,28]
	Feeding skills training	3	[23,24,46]
	Online-based dementia feeding skills training (Web, Mobile)	2	[15,48]
Sample size	≤50 persons	3	[15,26,48]
	51−100 persons	5	[25,27,28,29,46]
	>100 persons	2	[23,24]
Intervention length per session	≤60 min	5	[15,25,26,27,28]
	>60 min	4	[15,23,24,48]
Intervention duration	<8 weeks	4	[23,24,29,48]
	≥8 weeks	5	[15,25,26,27,28]

^1^ Group 1: Spaced retrieval (SR) training; Group 2: Montessori.

**Table 3:** Interventions related to eating behaviors in the intervention studies (*n* = 13).

Variable	Categories	*n*	Reference No.
Intrapersonal ^1^	Age	2	[35]
	Gender	2	[35,44]
	Comorbidities	1	[36]
	Duration of illness (dementia)	1	[7]
	Presence of dysphagia signs	1	[38]
	Number of medications	1	[37]
	Period of institutionalization	2	[7,40]
	Cognitive function	7	[7,8,36,37,38,39,42]
	Physical function	4	[7,8,35,37]
Interpersonal ^1^	Close relationship with family	2	[35,44]
	Caregiver assistant	1	[43]
Environmental ^1^	Diet type	1	[7]
	Eating place	2	[7,34]
	Presence of staff	3	[7,34,35]
	Length of the eating time	1	[41]
	Eating with other residents	1	[44]
	Physical (tablecloth, illuminance level, sound volume level, etc.)	4	[34,41,43,44]

^1^ Multiple choices.

## Data Availability

Not applicable.

## Supplementary Materials

The following are available online at https://www.mdpi.com/article/10.3390/ijerph181910109/s1, Table S1: Summary of included Studies (*n* = 39), Table S2: Search strategy used in each database.
