# Supplementary material for: Eating Difficulties among Older Adults with Dementia in Long-Term Care Facilities: A Scoping Review
Source: Int J Environ Res Public Health. 2021 Sep 26;18(19):10109. doi: 10.3390/ijerph181910109 (PMC8508293; doi:10.3390/ijerph181910109)
Supplement: Supplementary file 1 [file ijerph-18-10109-s001.zip › ijerph-1328617-supplementary.pdf]

Table S1. Summary of included Studies (*n* = 39)

Design	NO.	Author(s) (year)	Country	Aims	Population	Outcome measurements	Outcomes
Intervention study	A1	Chang et al. (2005)	Taiwan	To evaluate the effectiveness of a feeding skills training program for nursing assistants	▪ 67 nursing assistants<br>-exp. (n = 31)<br>-cont. (n =3 6)<br><br>▪ 36 nursing assistant–dementia patient dyads<br>-exp. (n =2 0)<br>-cont. (n = 16)	▪ The Formal Caregivers' Knowledge of Feeding Dementia Patient Questionnaire<br>▪ The Formal Caregivers' Attitude toward Feeding Dementia Patient Questionnaire<br>▪ The Formal Caregivers' Behaviors toward Feeding<br>▪ EdFED<br>▪ total eating time<br>▪ food intake	▪ **The Formal Caregivers' Knowledge of Feeding Dementia Patient Questionnaire**<br>: nursing assistants in the treatment group had significantly higher mean difference of knowledge scores than those in the control group (F=47.7, p < .001).<br>▪ **The Formal Caregivers' Attitude toward Feeding Dementia Patient Questionnaire**<br>: The treatment group had a more positive attitude toward feeding dementia patients than those in control group(F=15.75, p< .001)<br>▪ **The Formal Caregivers' Behaviours toward Feeding**<br>: The nursing assistants in the treatment group had a significantly better behaviour scores than those in the control group (t =6.0, P < .05).<br>▪ **EdFED**<br>The dementia patients in the treatment group had a significantly higher EdFED scores (more feeding difficulty) (t=2.1, P < .05) than those in the control group.<br>▪ **Total eating time**<br>The dementia patients in the treatment group had a significantly longer eating time (t=2.7, P < .05) than those in the control group.<br>▪ **Food intake**<br>There was no

						significant difference on food intake between the two groups<br>(t=.08, P=.49) after the training programme.
A2	Chang et al. (2006)	Taiwan	To evaluate the effectiveness of a feeding skills training program for nursing assistants	▪ 67 nursing assistants<br>-exp. (n = 31)<br>-cont. (n = 36)<br><br>▪ 36 nursing assistant–dementia patient dyads<br>-exp. (n = 20)<br>-cont. (n = 16)	▪ The Formal Caregivers' Knowledge of Feeding Dementia Patients Questionnaire<br>▪ The Formal Caregivers' Attitude toward Feeding Dementia Patients Questionnaire<br>▪ The Perceived Behavior Control Scale<br>▪ The Intention Scale<br>▪ The Formal Caregivers' Behaviors in Feeding Dementia Patients Observation Checklist<br>▪ feeding during mealtime was observed	▪ **The Formal Caregivers' Knowledge of Feeding Dementia Patients Questionnaire**<br>: Nursing assistants who received the feeding skills training program were significantly (P < .001) more knowledgeable after the intervention than those who did not receive the training.<br>▪ **The Formal Caregivers' Attitude toward Feeding Dementia Patients Questionnaire**<br>: There was no difference between the groups in attitude scores (P < .99).<br>▪ **The Perceived Behavior Control Scale**<br>There was no difference perceived behavior control scores(P < .85).<br>▪ **The Intention Scale**<br>Nursing assistants in the treatment group had significantly higher scores on intention frequency than those in the control group (P < .05). There was no difference between the groups on intention belief scores (P < .11)<br>▪ **The Formal Caregivers' Behaviors in Feeding Dementia Patients Observation Checklist**<br>**:** Nursing assistants who completed the feeding skills training program (M < .57, SD < .14) had significantly (P < .009) higher feeding behavior scores than those who did not participate (M <.32, SD < .07).<br>▪ **Feeding during mealtime was observed**

						All trained nursing assistants used their new feeding skills, such as giving patients more time to eat and those related to dealing with feeding problems.
A3	Lin et al. (2010a)	Taiwan	To evaluate the effectiveness of a training protocol (Spaced Retrieval and Montessori-based activities) in decreasing eating difficulty	▪ 85 residents with dementia<br>-exp. (SR) (n =3 2)<br>-exp. (Montessori) (n =2 9)<br>-cont. (n = 24)	▪ Chinese version of EdFED<br>▪ MNA (Mini-nutritional assessment)<br>▪ Observation (Eating time, Eating amount, Residents fed by caregivers, Physical assistance, Verbal assistance)	▪ **Chinese version of EdFED**<br>: The EdFED scores and assisting feeding scores for the SR and Montessori-based activity groups after intervention were significantly lower than that of the control group.<br>▪ **MNA(Mini-nutritional assessment)**<br>: MNA in the SR group was significantly higher than that of the control group, while MNA in the Montessori-based activity group was significantly lower than that of the control group.<br>● **Observation**<br>: the frequencies of physical assistance and verbal ssistance for the Montessori-based activity group after intervention were significantly higher than that of the control group
A4	Lin et al. (2011)	Taiwan	To evaluate the effectiveness of a Montessori intervention for improving eating ability and nutritional status	▪ 29 residents with dementia<br>-Montessori intervention sequence I (n = 15)<br>-Montessori intervention sequence II (n = 14)	▪ Chinese version of EdFED<br>▪ EBS<br>▪ MNA<br>▪ Observation (Self-feeding frequency, Self-feeding time, Verbal assistance, Physical assistance, Residents fed by caregivers)	▪ **Chinese version of EdFED**<br>The EdFED scores and physical assistance post-test results were less than the pre-test results for the Montessori intervention period ($p < .05$).<br>▪ **EBS**<br>The EBS scores post-test results were more than the pre-test results for the Montessori intervention period ($p < .05$).<br>▪ **MNA**<br>With the exception of the MNA post-test score being significantly less than the pretest in the routine activities period, no other significant differences were found for any other variables.<br>▪ **Observation**

						The self feeding frequency and time post-test results were more than the pre-test results for the Montessori intervention period (p < .05).
A5	Wu et al. (2014a)	Taiwan	To evaluate the long-term effects of the standardized and individualized spaced retrieval combined with Montessori-based activities on eating ability	▪ 61 residents with dementia<br>-exp. (SR/EL group) (n =3 2)<br>-cont. (SR-only group) (n = 29)	▪ The proportion of each meal consumed	▪ **The proportion of each meal consumed**<br>: the SR/EL group (n=31) intake increased by 14.6% with an effect size of 0.65, whereas the SR-only group.<br>intake only increased by 4.8% with an effect size of 0.47.
A6	Wu et al. (2014b)	Taiwan	To evaluate the effects of using accumulating cues in a spaced retrieval paradigm on recall performance, cognitive status, and food intake	▪ 90 residents with dementia<br>-exp. (Montessori-based group) (n = 25)<br>-exp. (Individualized group) (n = 38)<br>-cont. (n = 27)	▪ Chinese version of EdFED<br>▪ Eating amount<br>▪ Body weight	▪ **Chinese version of EdFED**<br>The Chinese-EdFED scores at the pre-test for the standardized group were significantly higher than those of the control group (F= 3.67, P =.03).<br>▪ **Eating amount**<br>The eating amount at the pretest for the standardized group were significantly lower than that of the control group (F = 6.63, P = .002).<br>▪ **Body weight**<br>The body weight at the pretest for the standardized group were significantly lower than that of the control group (F= 4.29, P = .017).
A7	Batchelor-Murphy et al. (2015)	USA	To evaluate the feasibility of a web-based	▪ 7 nursing assistant–dementia patient dyads<br>-exp. (n = 4)	▪ EdFED<br>▪ Time spent providing meal assistance	▪ **EdFED**<br>The EdFED scores for both groups increased from baseline to

				dementia feeding skills training program for nursing home staff	-cont. (n = 3)	▪ Meal intake	Week 8. In the intervention group, the average score increased from 7.0 to 8.7 on a 20 point scale; in the control group, the average score increased from 4.8 to 6.5 on a 20 point scale.<br>▪ **Time spent providing meal assistance**<br>Feeding assistance provided to residents in the intervention group increased, and more food was consumed. The control group decreased of feeding assistance and less food was consumed<br>▪ **Meal intake**<br>The average meal intakes for the intervention group more than doubled, while meal intakes for the control group decreased by more than half.
	A8	Hsu et al. (2016)	Taiwan	To evaluate the effectiveness of spaced retrieval for improving hyperphagia	▪ 97 residents with dementia<br>-exp. (n = 50)<br>-cont. (n = 47)	▪ Dementia Hyperphagic Behavior Scale<br>▪ Food intake<br>▪ BMI	▪ **Dementia Hyperphagic Behavior Scale**<br>The frequency of hyperphagic behavior and scale scores decreased in the experimental group but increased in the control group.<br>▪ **Food intake**<br>The average amount of food intake in the experimental group decreased but that in the post-test control group increased.<br>▪ **BMI**<br>The degree of difference between the experimental and control groups was not significant, the BMI in the experimental group decreased to within the normal range as compared with the pretest results.
Observational study	A9	Durnbaugh et al. (1996)	USA	To present the Feeding Behaviors Inventory, an instrument designed to	▪ 20 residents with dementia	▪ Feeding behaviors inventory	▪ **Feeding behaviors inventory**<br>A total of 571 behaviors were identified. The common problem mealtime behaviors were observed 10 behaviors. The less common behaviors were observed 7 behaviors.

			identify common mealtime feeding behaviors			
A10	Berkhout et al. (1998)	Netherlands	To investigate the cause of weight loss in nursing-home patients with dementia	▪ 514 residents above 65 years in nursing home<br>-existing residents: (n =2 50)<br>-newly admitted: (n = 264)	▪ Nurses recorded the most important difficulties in self-feeding (choosing food, bringing food to the mouth, chewing, and swallowing)	▪ Significant relationship between body weight and being able to choose food, bring it to the mouth and chew it.<br>▪ If was little or no change in feeding disabilities, the average body weight showed little or no change.
A11	Amella (1999)	USA	To predict how the quality of the interaction between care giver and care receiver influenced the proportion of food consumed	▪ 53 residents with late-stage dementia.	▪ Proportion of food consumed (weighing)	**▪ Proportion of food consumed (weighing)**<br>The CNA's ability to allow another person to control a relationship were most predictive of the variance in the proportion of food consumed.<br>The quality of the resident-CNA interaction accounted for 32% of the variance in the proportion of food consumed.
A12	Amella (2002)	USA	To investigate factors regarding resistance behavior at meals	▪ 53 residents with dementia<br>-resistors (n = 23)<br>-acceptors (n = 30)	▪ EdFED-Q<br>▪ BMI<br>▪ Proportion of food consumed (weighing)<br>▪ Time taken to assist with meals	When examining resistance to feeding a variety of factors must be examined, including the quality of the interaction between the caregiver and the person being fed.<br>**▪ EdFED-Q**<br>Two groups emerged from the sample of 53 dyads as identified by their behavior rating on the EdFED-Q: 23persosns were categorized as "resistors" and 30 persons were "acceptors". There were significant differences between

						resistors and acceptors on items 3-10 of the EdFED-Q.<br>▪ **BMI**<br>BMI didn't vary significantly with in the resistors and the acceptors.<br>▪ **Proportion of food consumed (weighing)**<br>The proportion of food consumed were significantly different in two groups with resistors consuming 51% and acceptor consuming 72%.<br>▪ **Time taken to assist with meals**<br>The time taken to assist with meals were significantly different in two groups with resistors taking 18minutes for assistance while acceptors took 13.8 minutes.
A13	Reed et al. (2005)	USA	To investigate factors associated with low food and fluid intake	▪ 421 residents with dementia	▪ The Structured Meal Observations (SMO)	▪ **The Structured Meal Observations (SMO)**<br>54% of observed residents had low food intake, and 51% had low fluid intake. Staff monitoring of residents, having meals in a public dining area, and the presence of noninstitutional features were each associated with higher food and fluid intake.
A14	Lin et al. (2010b)	Taiwan	To investigate the risk factors of low food intake	▪ 177 residents with dementia in LTC	▪ Chinese version of EdFED<br>▪ BMI	▪ **Chinese version of EdFED**<br>The mean of the EdFED for all participants was 2.42(SD2.82). For subsequent analysis, the median of 2 was used as cut-off point to be classified as having an eating difficulty because the normality of the EdFED was rejected(Kolmogorov-Smirnov=0.293,p-0,00).<br>▪ **BMI**<br>There was no significant difference between BMI & Low food intake.
A15	Slaughter et al. (2011)	Canada	To estimate the incidence and identify the predictors of	▪ 120 nursing home residents	▪ Researchers observed residents' loss of eating ability	▪ 49 residents were observed to experience an episode of eating disability within one year, of whom 25 had at least one episode of excess disability in eating.

			eating disability due to dementia		during meals; eating disability was defined as receiving physical assistance to put food into the mouth or not eating at all	▪ The Kaplan-Meier estimates of eating disability and excess disability in eating were 40.8%(95%CI:32.7%-5.2%)and 23.5%(95%CI: 16.6%-33.0%)respectively.<br>▪ More than one half ot the eating disability was due to something other than dementia, namely excess disability.
A16	Chang et al. (2012)	Taiwan	To investigate factors associated with feeding difficulty of individuals with dementia	▪ 93 residents with dementia	▪ Chinese version of EdFED	▪ **Chinese version of EdFED**<br>Residents with EdFED scores above 5 had significantly longer duration of dementia, greater dependence, lower BMI, greated cognitive impairment than those with EdFED scores under 5.
A17	Edahiro et al. (2012)	Japan	To investigate factors affecting self-feeding	▪ 150 Alzheimer's disease patients who were hospitalized in dementia ward	▪ Feeding Cycle Recording<br>▪ Eating-related BPSD item	▪ **Feeding Cycle Recording**<br>A strong relationship was observed between rate of independence in eating and MMSE score.<br>▪ **Eating-related BPSD item**<br>The following items were significantly more frequently oberved as the severity of dementia increased.: "difficulty in rinsing/gargling", "presence of dysphagia signs", "difficulty in beginning a meal", "difficulty in proper use of utensils", "difficulty in scooping the proper amount of food", "difficulty in recognizing the total amount of food provide", "difficulty in maintaining alertness while eating".
A18	Hanson et al. (2013)	USA	To describe quality of care for feeding problems in	▪ 256 residents with dementia	▪ Quality of feeding assistance provided by staff<br>▪ Body weight loss	▪ **Quality of feeding assistance provided by staff**<br>73% of residents had difficulty taking adequate food and water. Most surrogates felt very

			residents with advanced dementia, and probability and predictors of weight loss and mortality.		▪ Mortality	involved in decisions about the residents nutrition. 23% of family caregivers felt the residnet with dementia was getting less feeding assistance than needed.<br>▪ **Body weight loss**<br>9-11% had significant weight loss during each 3-month inverval of follow-up.<br>▪ **Mortality**<br>Weight loss at enrollment was the only independent predictor of mortality.
A19	Wu (2014)	Taiwan	To explore the prevalence and predictors of hyperphagic behaviors	▪ 179 residents with dementia	▪ Hyperphagia questionnaire<br>▪ Cognitive abilities screening instrument<br>▪ Cohen-Mansfield agitation inventory<br>▪ Demographic characteristics	▪ **Hyperphagia questionnaire**<br>The most commonly experienced hyperphagic behavior was eating continually in the presence of food (mean = 1.6, SD = 0.9), followed by eating when seeing food(mean=1.1, SD =0.9) and eating quickly when seeing food (mean = 1.1, SD =1.1).<br>▪ **Cognitive abilities screening instrument**<br>The significant predictor of hyperphagia was category fluency(poor category fluency ability(B = j0.38, p = .002, OR =0.68, 95%CI [0.54, 0.87])<br>▪ **Cohen-mansfield agitation inventory**<br>The significant predictors of hyperphagia were more physically nonaggressive behavior subscale scores(B = 0.12, p = .009, OR = 1.13, 95% CI [1.03, 1.23]).<br>▪ **Demographic characteristics**<br>The significant predictors of hyperphagia were gender and length of institutionalization(male participants(B = 0.86, p =.01, OR = 2.37, 95% CI [1.21, 4.63] , longer institutionalization (B =0.14, p =.02, OR = 1.14, 95%CI [1.02, 1.28]).

						There were significant differences between the two groups(with and without hyperphagia) in terms of gender, length of institutionalization, and CASI and CMAI scores (22 = 6.16, p =.01; t = j2.50, p =.01; t =2.33, p =.02;and t = j3.08, p = .002, respectively).
A20	Lee et al. (2015)	Korea	To investigate factors associated with eating ability	▪ 149 residents with dementia	▪ MMSE-K<br>▪ Korean activities of daily living scale<br>▪ Eating Behavior Scale	**▪ MMSE-K**<br>The mean score of cognitive function was 9.11, indicating that most of the participants had severe dementia<br>**▪ Korean activities of daily living scale**<br>The mean score of physical function was 17.33, indicating that most of the participants had severe dependence for physical function.<br>**▪ Eating Behavior Scale**<br>The mean score of the EBS was 10.43 (range, 1–18). The highest score was obtained for the ability to bite, chew, and swallow without choking (mean = 2.36, SD = 0.86), and the lowest score was obtained for the ability to appropriately use utensils (mean = 1.11, SD = 0.95; Table 2).<br><br>There were significant differences in eating ability according to the participants' general characteristics, such as duration of residence(lowest for a duration of 3–4 years) (F = 3.129, p = 0.005), duration of illness(a longer duration of illness had a lower EBS score)(F = 17.242, p < 0.001), degree of visual impairment(lower when the degree of visual impairment was classified as "impossible to judge the objective" rather than normal or impaired. (F = 6.752, p < 0.001), eating place(lower when their eating place was a room other than a living room or dining room)(F = 20.727, p < 0.001), and diet type(higher in

						participants on a general diet rather than a soft and liquid diet)(F = 26.230, p < 0.001; Table 3).<br><br>The level of dependence of participants with respect to eating ability was correlated with cognitive function (r = 0.740, p < 0.001) and physical function (r = 0.666, p < 0.001).<br><br>Cognitive function, physical function, duration of illness, eating place (living room or public eating place) and diet type (soft diet or liquid diet) significantly (b = 0.331, 0.245, 0.223, 0.197 and 0.145, respectively; p < 0.05) predicted the eating ability of the participants (R^2 =0.722; F= 52.399; p < 0.001).
A21	Liu et al. (2016)	USA	To investigate the association between specific personal and environmental factors and eating performance	▪ 199 residents with dementia	▪ Using the single self-care ‘feeding’ item in the Barthel Index<br>▪ MMSE<br>● Using the single ‘chair sit-sitting balance’ item in the Tinetti Gait and Balance scale<br>▪ Physical Capability Scale (PCS)<br>▪ Cornell Scale for Depression in Dementia (CSDD)<br>▪ Cohen-Mansfield Agitation Inventory-short form (CMAI)	**▪ Using the single self-care 'feeding' item in Barthel Index.**<br>Almost one-third (n= 64, 32.2%) of the 199 residents needed help with eating performance.<br><br>Compared with LTC residents with severe cognitive impairment, those with moderate impairment were 1.7 times more likely to demonstrate independence in eating performance.<br><br>**▪ MMSE**<br>The majority received high school education (n= 91, 45.7%) had severe cognitive impairment (n= 125, 62.8%).<br><br>Cognitive impairment (OR = 2.70, 95% CI: 1.24-5.87) and physical capability (OR = 1.13, 95% CI: 1.01-1.28) were significantly associated with eating performance.

						▪ **Using the single 'chair sit-sitting balance' item in the Tinetti Gait and Balance scale.**<br>The majority received high school education (n= 91, 45.7%), were unable to sit in the chair independently (n= 104, 52.3%).<br><br>▪ **Physical Capability Scale (PCS)**<br>As physical capability increased by one unit on the PCS, the likelihood of demonstrating independence in eating among LTC residents increased by 13%.<br><br>▪ **Cornell Scale for Depression in Dementia (CSDD)**<br>▪ **Cohen-Mansfield Agitation Inventory-short form (CMAI)**<br>Neither depressive symptoms nor agitation was associated with eating performance.
A22	Chang et al. (2017)	Taiwan	To identify the best cutoff point for the Chinese Feeding Difficulty Index (Ch-FDI) and factors associated with eating behaviors	▪ 213 residents with dementia	▪ Chinese Feeding Difficulty Index<br>▪ EdFED	▪ **Chinese Feeding Difficulty Index**<br>The prevalence of food intake difficulties at lunchtime (Ch-FDI-Lunch ≥5) and dinnertime (Ch-FDI-Dinner ≥4) were 36.2% and 42.3%, respectively.<br><br>The overall prevalence of food intake difficulties was 44.6% (Ch-FDI ≥4)<br><br>Food intake difficulties during lunchtime were significantly correlated with cognition (r = -0.201, p = 0.003), independence in ADLs (r = -0.181, p = 0.008), the ADL-Q1 (r = -0.231, p = 0.001), BMI (r = -0.137, p = 0.046), and length of eating time (r = 0.391, p<0.001).<br><br>Food intake difficulties during dinnertime were significantly correlated with cognition (r = -0.167, p = 0.015) ADL-Q1 (r = -0.143, p = 0.037),

						the illuminance level (r = -.0193, p = 0.005), sound volume level (r = 0.146, p = 0.033), and eating time (r = 0.339, p<0.001).<br>▪ **EdFED**<br>The prevalence of food intake difficulties (EdFED≥5) was 43.66%, using a cutoff point of 5 based on Chang's (2012) study.
A23	Maria Perez-Sanchez et al. (2018)	Spain	To evaluate the relation between altered eating behaviors/attitudes and nutritional status	▪ 139 residents with severe cognitive impairment	▪ The Blanford's Aversive Feeding Behaviours Inventory<br>▪ The 26-item Eating Attitudes Test<br>▪ Dietary intake<br>▪ MNA	▪ **The Blanford's Aversive Feeding Behaviours Inventory**<br>AFBI score was significantly higher in those subjects with malnutrition.<br><br>An inverse relation was observed between energy, carbohydrates, lipid intakes and AFBI score.<br><br>Those subjects with severe cognitive impairment presented a significantly higher score in the AFBI (p <0.001), which reflects inadequate dietary attitudes as cognitive status get worse.<br>▪ **The 26-item Eating Attitudes Test**<br>No subject showed a score indicating the presence of symptoms associated with classic eating disorders such as anorexia or bulimia (>20 points).<br><br>An inverse relationship between the EAT-26 score and the number of lymphocytes (r = -0.314, p = 0.049) was observed.<br>▪ **Dietary intake**<br>Textures of diets were adapted to the patients' needs. Although most of the subjects ate a

						normal diet (62%), 21% of the subjects ate an easy mastication diet and the remaining 17% followed a semisolid (puree) diet.<br>▪ **MNA**<br>33.1% of the population was malnourished. Those subjects with lower MNA score had higher EAT-26 score, particularly regarding the oral control scale.
A24	Liu et al. (2018)	USA	To examine the association of resident characteristics, staff mealtime assistance, and environmental stimulation with the pace of food intake	▪ 19 NA and 15 residents with dementia	▪ The pace of food intake recorded on video<br>▪ Level of Eating Independence (LEI) scale<br>▪ Staff mealtime assistance recorded on video	A faster pace of food intake is associated with being male, better eating performance, staff provision of visual and physical assistance and better quality of environmental stimulation that involved interaction.
A25	Palese et al. (2020a)	Italy	To explore the influence of nursing home environment on eating independence	▪ 1027 residents with dementia	▪ Italian-validated version of EdFED	▪ **Italian-validated version of EdFED**<br>At the resident level, the following variables reduced the likelihood of eating dependence: a higher Barthel Index score (b= 2.513, p=<0.001); eating in the dining room surrounded by other residents (b= 1.968, p=<0.006) or near two residents (left plus right [b= 1.615, p=<0.001]; left/right plus in front [b= 1.333, p = 0.002]) compared with eating alone in the bedroom, and having a close relationship with family (b= 0.850, p = 0.006).

							However, female residents (b=0.777, p = 0.006) had an increased risk of eating dependence.<br><br>At the nursing care level, the number of environmental interventions (b=0.579, p=<0.001) increased the risk of eating dependence, while the number of interventions the nursing staff performed daily at the resident level was negatively associated with eating dependence (b= 0.338, p=<0.001).<br><br>At the NH unit environment level, those facilities where the TESS-NH score was low (<110), the NH environmental status worsened the eating dependence score, while on the other hand, a higher TESS-NH score had a protective effect on eating dependence, especially for residents with a CPS ≥4.
Qualitative study	A26	Athlin et al. (1989)	Sweden	To understand feeding problems in patients with severe dementia cared for in a task assignment system	▪ 15 patients with severe dementia and 45 caregivers who fed the 15 patients during the study period	▪ Researchers analyzed video-recorded meals for patients with severe dementia and interviews with caregivers who assist them.	▪ “Task aspects”<br>When the patients were video-recorded during two meals, 40 out of the 54 problems noted (74%) were seen in both meals.<br><br>The result from the interviews showed that the agreement between different caregivers’ opinion of the problems of the same patient varied from 17 to 100% (m=52%).<br><br>Problems mentioned in the interviews that could not be seen in the video analysis<br>: Is “time-consuming’’(n=9), Has a posture /movements that hinders(n=8)<br><br>“relationship aspects”<br>In the video-recordings, description of the feeding problems

							: Unsatisfactory synchrony between caregiver and patient(n=12), the caregiver's behaviour is an obstacle(n=10)<br><br>In the interviews, description of the feeding problems<br>: Difficult to establish contact with the patient(number of interviews/patients = 44/12, agreement between caregivers' opinion 90%), Difficult to interpret the patient's cues(number of interviews/patients = 40/11, agreement between caregivers' opinion 84%)
Mixed methods	A27	Chang et al. (2008a)	Taiwan	To investigate factors related to feeding difficulty that are shown in the interaction between nursing assistants and residents	▪ 48 residents with dementia and 31 nursing assistants	▪ Chinese version of EdFED<br>▪ Nursing assistant interview about feeding dementia residents	▪ **Chinese version of EdFED**<br>The most frequent feeding problem was refusal to eat from "sometimes" to "often" (n=18). No residents left their mouths open during eating or allowed food to drop out of the mouth.<br><br>Additional problems observed during feeding were pushing away the caregiver or tray (n=2), being unable to sit still (n=2), hitting the tray or caregiver (n=1), and protruding the lips (n=2). Some residents were drowsy or had difficulty waking up (n=1), while others became angry (n=4) or distracted (n=3) while they ate. A few choked and had swallowing difficulties (n=3) or verbally refused to eat (n=18), consistently pushed away (n=2), or would not open their mouths (n=8).<br>▪ **Nursing assistant interview about feeding dementia residents**<br>Nearly all nursing assistants (93%) reported that feeding residents with dementia was time-consuming and that they lacked enough staff and knowledge to feed residents well.

	A28	Shinagawa et al. (2016)	Japan	To develop a possible classification of eating-related problems	▪ 208 residents and patients	▪ Semi-structured systematic interviews with nurses stationed on Eating and Swallowing<br>▪ Mini-Mental State Examination (MMSE)<br>▪ Clinical Dementia Rating (CDR)<br>▪ Neuropsychiatric Inventory (NPI)	▪ **The factor analysis (24items) for eating-related problems in dementia**<br>Factor 1 (overeating) was not related to independent variables; Factor 2 (swallowing problems) was related to BMI (β=.82, P < .05), MMSE (β=.94, P < .05) and CDR scores (β=1.56, P < .05); Factor 3 (decrease in appetite) was related to NPI score (β=4.51, P < .05); Factor 4 (obsession with food was related to MMSE (β=.90, P < .05), NPI (β=2.21, P < .05).
	A29	Jung et al. (2020)	Korea	To assess the feasibility and examine the preliminary effectiveness of a mobile application-based meal assistant training program for use by direct care workers	▪ 23 older adults with dementia-caregiver dyads	▪ Eating behavior scale (EBS)	▪ The effects of the app intervention on the older adults with dementia and their caregivers were not significant; however, the findings from focused group interviews support the potential usefulness of the app intervention for long-term care workers who need meal assistant skills education.
Literature review	A30	Keller et al. (2006)		To present meal rounds as a potential intervention for identifying nutrition	▪ a previously published study on 37 residents	N/A	▪ **Description of Meal Rounds**<br>“Meal rounds” involve the brief and informal observation of residents in their dining environment while they are eating or being fed a meal.

			problems and specifically feeding, food texture, and mealtime behaviors.			Specific areas of concern are Admission Diet Prescription, Proportion and Types of Food Consumed, Dysphagia, Difficulty Using Utensils, Positioning for Meals, Disruptive Behavior, Extreme Fatigue During Meals, Slow to Eat, Hyperphagia, Feeding Assistance, Resists Assistance at Meals, Agitation.<br>▪ **Standardized Eating Behavior Scales for Meal Rounds**<br>Edinburgh Feeding Evaluation in Dementia Scale(EdFed), Eating Behavior Scale(EBS)<br>▪ **Outcomes: The importance of meal rounds**<br>The prevalence of eating and mealtime behaviors or issues and their association with nutrition risk demonstrates the potential importance of meal rounds as an intervention for prevention of weight loss and undernutrition.
A31	Cleary (2007)	Canada	To review the current approaches to manage feeding and swallowing disorders	N/A	N/A	▪ **Deficits and strengths of people with ADOD** :<br>Reduce distractions; Train and supervise staff in predictable dining room routines; Implement individualized dining plans based on comprehensive feeding/swallowing assessments.<br>▪ **Adequate staffing** : Management and operational staff ensure adequate staffing levels and provide regular ongoing supervision and assistance with mealtimes.<br>▪ **Assessments** : The need for individualized and comprehensive assessments by skilled clinicians<br>▪ **Swallowing disorders** : Dysphagia diet programs; Frazier water protocol; Therapeutic dining and swallowing programs

A32	Aselage et al. (2011)	USA, UK, Australia	To explore the state of mealtime difficulties; characteristics, measurements, related factors, and interventions for alleviating mealtime difficulties	N/A	N/A	▪ **Characteristics** :refusing to eat, spitting out food, refusing to swallow, leaving food in the mouth, and/or turning their head away<br>▪ **Measurements** : Body weight, BMI, meal intake, diet orders, medication reviews, MMSE, functional status scales, EdFED and/or SMO tool<br>▪ **Related factors**:the quality of the interaction; the amount of time taken by the CNAs to assist with feeding; institutional factors (eg, short staffing, task-oriented institutional norms vs resident-oriented institutional norms) ; the characteristics of the resident (eg, severity of dysphagia, resident values and norms); physical, social, and/or psychological etiologies; the frequency of family visitation<br>▪ **Interventions for alleviating mealtime difficulties** : Clinical practice guidelines; a comprehensive feeding skills training program
A33	Chang et al. (2011)	Taiwan, USA	To propose strategies for feeding patients that caregivers can use	N/A	N/A	**Proposing strategies for feeding patients with dementia**<br>▪ **Assessing feeding difficulties** : Three instruments commonly used to measure feeding difficulties in patients with dementia : EdFED, the Feeding Behaviors Inventory, and EBS.<br>▪ **Contributing factors** : The cognitive, physical, psychological, social, environmental, and cultural factors that can contribute to, reduce, or prevent these difficulties.<br>▪ **System solutions** : The system level factors are best managed in social policies and environmental design. Institutional policies that promote family involvement in feeding and social interaction between patients and caregivers should be encouraged. Small changes(lightning, utensils, avoiding

							overcrowding) in the dining environment can support self-feeding behaviors.<br>▪ **Nursing interventions** : Five general types of feeding difficulties(initiating the feeding, maintaining attention, getting food into the mouth, chewing food, and swallowing food) are identified. The observable behavior associated with each, and the multidisciplinary and feeding strategies are addressed.<br>▪ **Allocating personnel** : Health care settings should provide an adequate number of well-trained personnel for feeding assistance, and maintain consistency between feeders and patients.<br>Monitoring outcomes: Inadequate food intake, weight loss, malnutrition, aspiration, and pulmonary complications are adverse outcomes associated with feeding difficulties.
A34	Cole (2012)	USA	To explore interventions that can be undertaken to establish and maintain adequate nutritional intake	▪ 12 articles	N/A		▪ **Causes of low food intake**<br>lack of independent feeding; depression; the loss of appetite and a change in preference towards sweet foods; lack of staff training; frequent family visits<br>▪ **Nutritional supplements:** increase in body weight; those with a low BMI tended to eat less food for lunch after being given a supplement in the morning: weak evidence in adequate nutritional intake.<br>▪ **Staff training programmes**<br>Increased staff competence promoted adaptation to individual feeding situations and staff were better able to adjust the eating environment to meet the needs of older people with dementia; The mealtime environment on the intervention ward improved(tablecloths, curtains and flowers were provided and the staff no longer wore uniforms); An improved menu and were monitored by the dietician

Systematic review	A35	Chang et al. (2008b)	Taiwan	To use concept analysis to identify characteristics of feeding difficulty and its antecedents and consequences that provide direction for assessment and management	▪ 71 articles	N/A	▪ Not only is getting food into the mouth important but also effective chewing and swallowing are necessary to transport food into the stomach without aspiration into the lungs or dribbling from the mouth.<br>▪ Feeding difficulty of older adults with dementia is a multidimensional phenomenon that is within the purview of nursing care<br>▪ Feeding difficulties arise at the interface between the caregiver strategies to assist the older adult with getting food into the mouth and chewing and swallowing food.
	A36	Liu et al. (2014)	USA	To evaluate the effectiveness of interventions on mealtime difficulties	▪ 22 intervention studies<br>-2082 residents with dementia<br>-95 professionals<br>-85 long-term care facilities	N/A	● **Nutritional supplements**: Moderate evidence to increase food intake, body weight and BMI.<br>● **Training/education programs**: Demonstrated moderate evidence to increase eating time and decrease feeding difficulty. Insufficient to increase food intake.<br>● **Feeding assistance:** Insufficient to increase food intake.<br>● **Environment/routine modification**: Indicated low evidence to increase food intake, and insufficient to decrease agitation.<br>● **Nnutritional status, eating ability, behavior disturbance, behavioral and cognitive function, or level of dependence**: Evidence was sparse
	A37	Liu et al. (2015)	USA	To evaluate the effectiveness of interventions	▪ 11 articles	N/A	● **Training programs targeting older adults** (Montessori methods and spaced retrieval) :good evidence in decreasing feeding difficulty.

			on eating performance			● **Mealtime assistance offered by nursing staff** (e.g., verbal prompts and cues, positive reinforcement, appropriate praise and encouragement) : effectiveness in improving eating performance.<br>● **Environmental modification**: lighting and table setting contrast on eating performance of residents with weak quality<br>● **Multicomponent intervention**: family style meal; staff training on prompting and praising appropriate mealtime behaviors improved residents' participation in eating tasks; intervals of appropriate communication; familiar physical activity; music maintained eating performance.
A38	Fetherstonhaugh et al. (2019)	Australia	To review the literature on strategies for promoting mealtime function in people with dementia living in residential aged care facilities and assess their effectiveness	▪ 20 articles	N/A	● **Interventions designed to improve mealtime function** : strategies related to the way in which food was presented, meal styles with respect to seating and serving arrangements, adaptations to the dining environment, mealtime skills training for people with dementia, music therapy, animal-assisted therapy<br>● **Impact of interventions on mealtime function :** Effects of interventions designed to train people with dementia or care staff in mealtime skills, primarily reported a positive impact of the interventions on food intake.<br>●**Outcomes related to mealtime behavior:** communication skills, increased lighting in the dining area, family style meals, a usual dining room experience with pre-plated meals, playing quiet relaxation music<br>● **Outcomes related to mealtime specific skills:**<br>The effects of the Montessori program or the spaced retrieval program differed according to the literature. Training of care staff was also

							associated with longer eating times, but no difference in overall food intake
Scoping review	A39	Palese et al. (2020b)	Italy, U.K.	To map the state of the research designed to maintain and/or promote independent eating	▪ 17 articles	N/A	●**Conceptual frameworks**: Conceptual frameworks supporting interventions' effectiveness have rarely been described in previous studies.<br>●**Intervention**: Studies have moved from simple, direct interventions to more complex interventions, as Montessori or specific education training programs.<br>●**Outcomes**: Eating performance (EdFED, eating time, amount of time staff dedicate to assist residents); Clinical outcomes (weight, food consumed, BMI, MNA; Adverse events

exp. = experiment; cont. = control; NA = Nurse assistant EdFED = Edinburgh Feeding Evaluation in Dementia; BMI = Body Mass Index; EBS = Eating Behavior Scale; MNA = Mini-nutritional assessment; MMSE = Mini-Mental State Exams; ADOD = Alzheimer's Disease and Other types of Dementia; NPI = Neuropsychiatric Inventory; SMO =

the Structured
Meal Observation
tool; CDR =
Clinical Dementia
Rating

Table S2. Search strategy used in each database

PubMed

	Searches	Results
#1	"dementia"[MeSH Terms] OR "dementia"[All Fields] OR "dementias"[All Fields] OR "dementia's"[All Fields]	227,382
#2	"geriatric"[All Fields] OR "geriatrics"[MeSH Terms] OR "geriatrics"[All Fields] OR "aged"[MeSH Terms] OR "aged"[All Fields] OR "older"[All Fields] OR "olders"[All Fields] OR "elderly"[All Fields] OR "elderlies"[All Fields] OR "elderly s"[All Fields] OR "elderlys"[All Fields] OR "senior*"[All Fields] OR "geriatric*"[All Fields]	5,898,671
#3	"eating"[MeSH Terms] OR "eating"[All Fields] OR "feeding"[All Fields] OR "feedings"[All Fields] OR "feeds"[All Fields]	427,190
#4	"facilit*"[All Fields] OR "long term"[All Fields] OR "institutional*"[All Fields] OR "nursing home*"[All Fields]	1,859,626
#5	#1 AND #2 AND #3 AND #4	574
#6	#5 Additional limits – Language : English, Date: December 31, 2020	**520**

Cochran

	Searches	Results
#1	MeSH descriptor: [Dementia] explode all trees	6105
#2	(dementia):ti,ab,kw	13,317
#3	#1 OR #2	15,571
#5	MeSH descriptor: [Geriatrics] explode all trees	206
#6	MeSH descriptor: [Aged] explode all trees	211,221
#7	(Geriatrics OR Aged OR aged OR older OR olders OR elderly OR elderlies OR elderly s OR elderlys OR senior* OR geriatric*):ti,ab,kw	562,734
#8	#5 OR #6 OR #7	562,734
#9	MeSH descriptor: [eating] explode all trees	3,639
#10	("eating" OR "feeding" OR "feedings" OR "feeds"):ti,ab,kw	35,190
#11	#9 OR 10	35993
#12	("facilit*" OR "long term" OR "institutional*" OR "nursing home*"):ti,ab,kw	111,151
#13	#3 AND #8 AND #11 AND #12	**66**

PsycINFO via ProQuest

	Searches	Results
#1	Thesaurus: [Dementia] explode major	41,624
#2	(dementia):Anywhere	82,749
#3	#1 OR #2	82,749
#4	Thesaurus: [Geriatrics] explode major	13,185
#5	Thesaurus: [Aged] explode major	218,533
#6	(aged OR older OR elderly OR senior* OR geriatric*):Anywhere	2,176,235
#7	#4 OR #5 OR #6	2,176,235
#8	Thesaurus: [Eating Disorders] explode major	17,124
#9	Thesaurus: [Eating Behavior] explode major	14,230
#10	Thesaurus: [Eating Attitudes] explode major	1,631
#11	("eating" OR "feeding" OR "feedings" OR "feeds"):Anywhere	108,464
#12	#8 OR #9 OR #10 OR #11	108,464
#13	"facilit*" OR "long term" OR "institutional*" OR "nursing home*":Anywhere	427,350
#14	#3 AND #7 AND #12 AND #13	270
#15	#20 Additional limits - Language: English, Date: December 31, 2020	**256**

CINAHL via EBSCO

	Searches	Results
#1	MH dementia OR AB dementia OR AB dementias OR AB dementia's	62,347
#2	MH Geriatrics OR MH Aged OR AB aged OR AB older OR AB olders OR AB elderly OR AB elderlies OR AB elderly s OR AB elderlys OR AB senior* OR AB geriatric*	1,053,623
#3	MH eating OR AB eating OR AB feeding OR AB feeds OR AB feedings	67,308
#4	AB "facilit*" OR AB "long term" OR AB "institutional*" OR AB "nursing home*"	355,505
#5	#1 AND #2 AND #3 AND #4	233
#6	#7 Additional limits – Language : English, Date: December 31, 2020	**228**
